# Supplementary material for: Targeting tankyrase scaffolding in the β-catenin destruction complex by PROTAC overcomes the limitation of catalytic inhibitors in colorectal cancer
Source: eLife. 2026 Nov 4;14:RP109052. doi: 10.7554/eLife.109052 (PMC13734679; doi:10.7554/eLife.109052)
Supplement: Supplementary file 2 [file elife-109052-fig1-data1.zip › Figure 1-source data 1. Original files for western blot analysis displayed in Figure 1B.pdf]

Fig1b_1_IWR1_TNKS

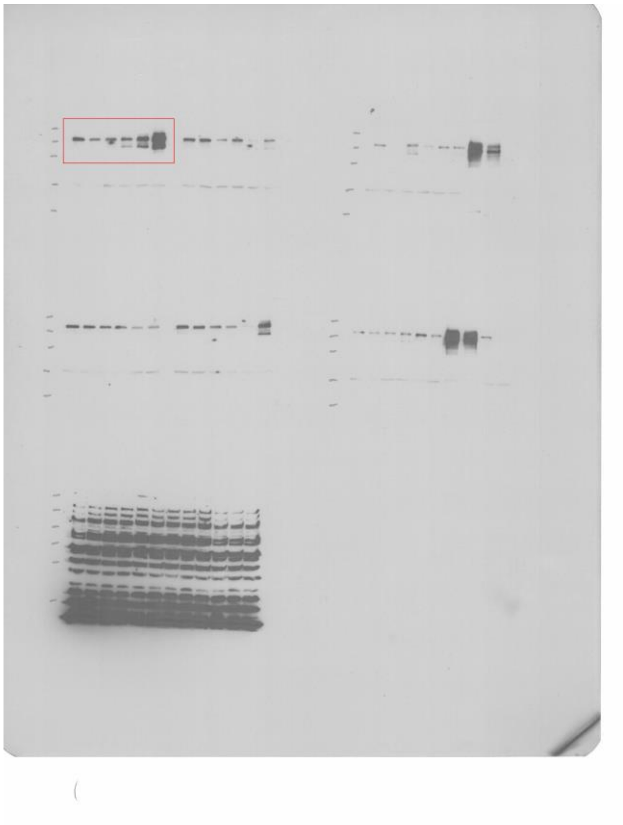

Fig1b_2_IWR1_GAPDH

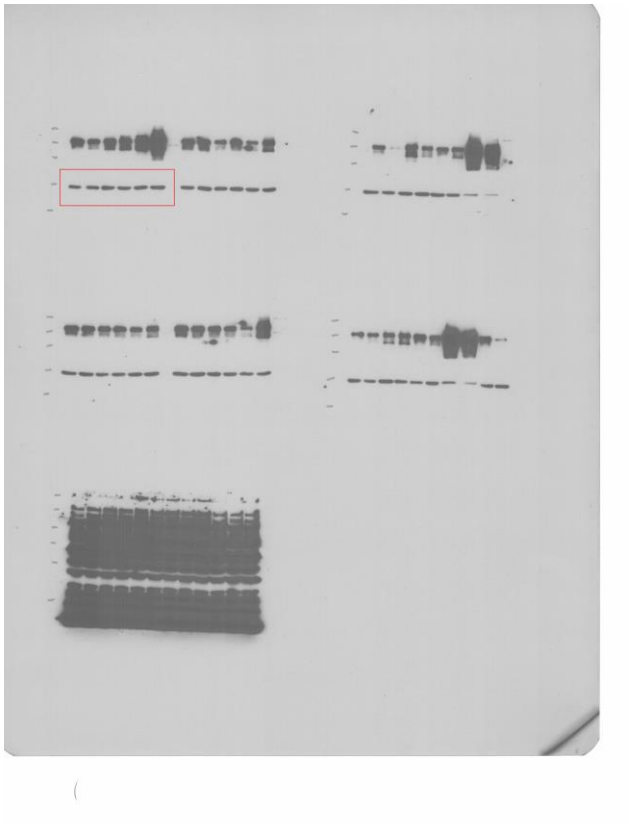

Fig1b_3_IWR1POMA_TNKS

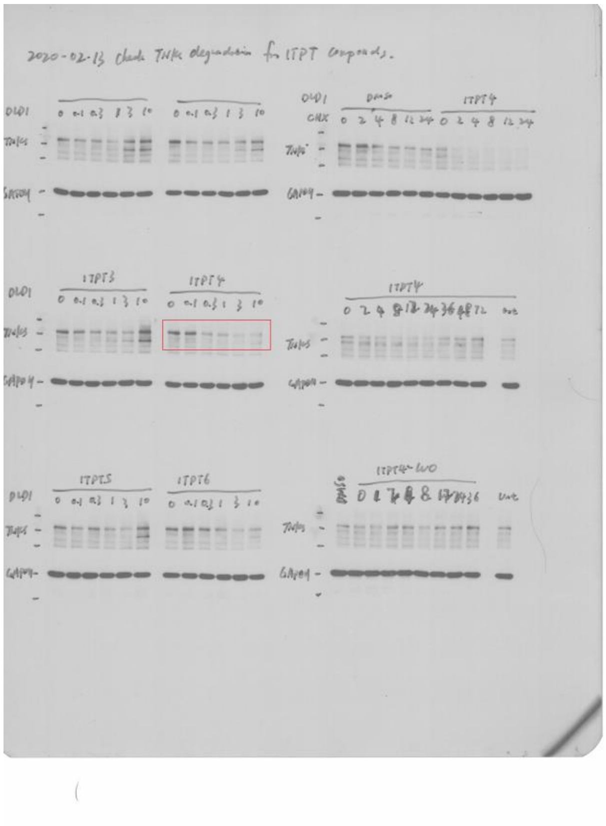

Fig1b_4_IWR1POMA_GAPDH

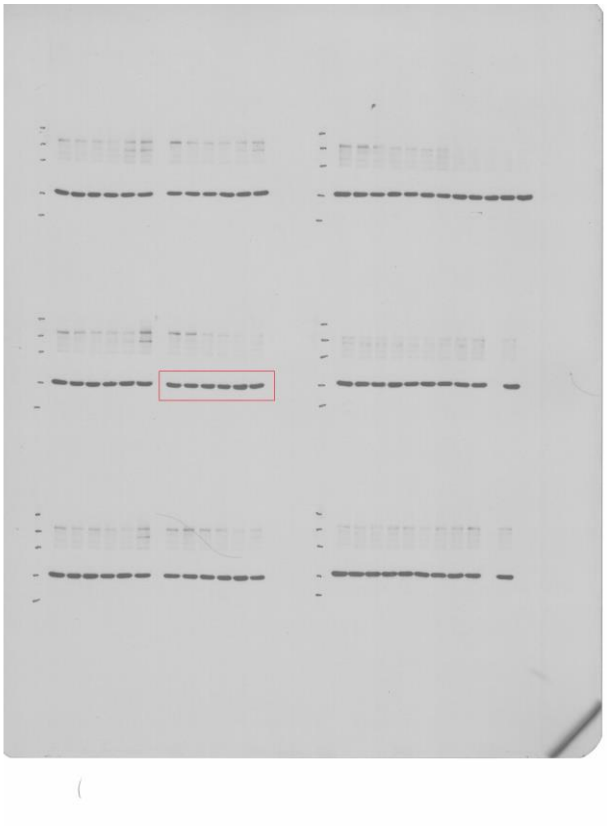

Figure 1. Characterization of IWR1-POMA. (B) IWR1 induced massive TNKS accumulation while IWR1-POMA promoted deep degradation in a dose and time-dependent manner in DLD-1 cells.
