# Supplementary material for: Targeting tankyrase scaffolding in the β-catenin destruction complex by PROTAC overcomes the limitation of catalytic inhibitors in colorectal cancer
Source: eLife. 2026 Nov 4;14:RP109052. doi: 10.7554/eLife.109052 (PMC13734679; doi:10.7554/eLife.109052)
Supplement: Supplementary file 2 [file elife-109052-fig1-data1.zip › Figure 1-source data 3. Original files for western blot analysis displayed in Figure 1D_1.pdf]

Fig1d_1_TNKS

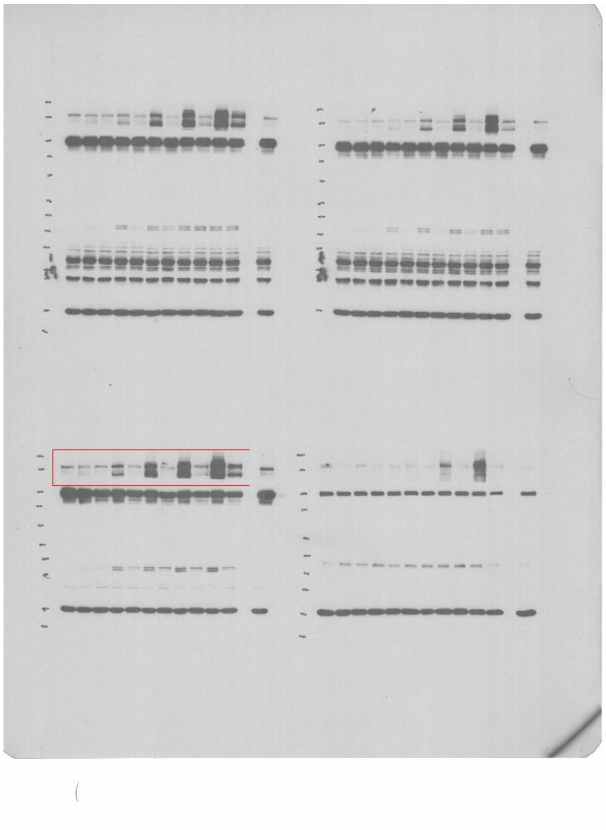

Fig1d_2_AXIN1

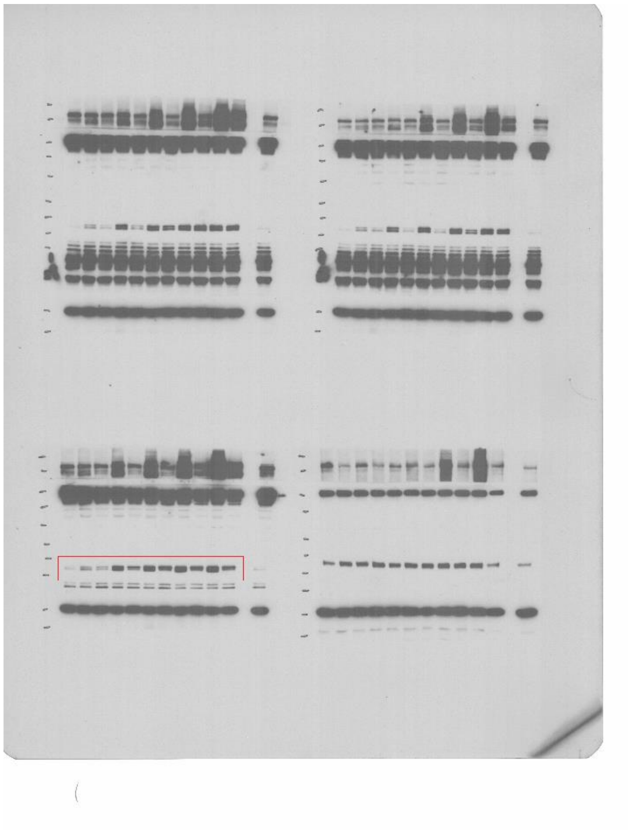

Fig1d_3_CTNNB1

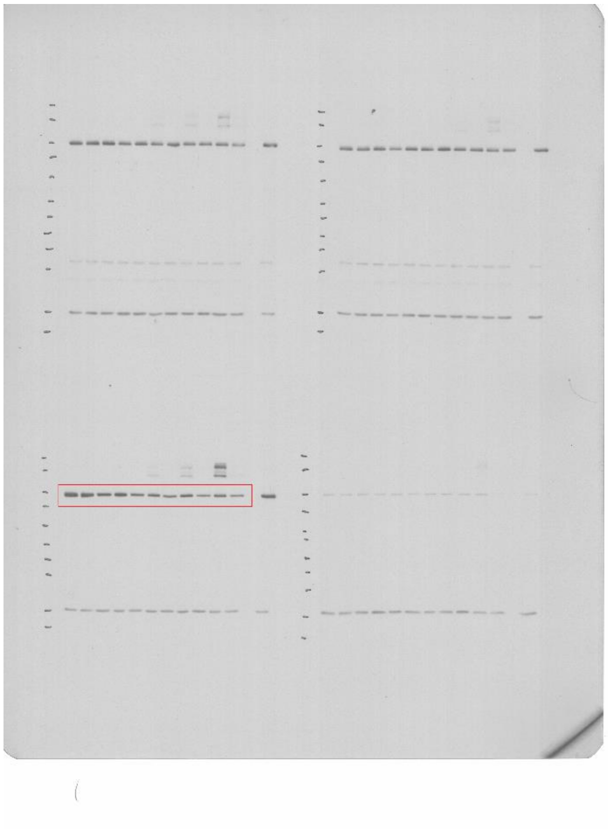

Fig1d_4_GAPDH

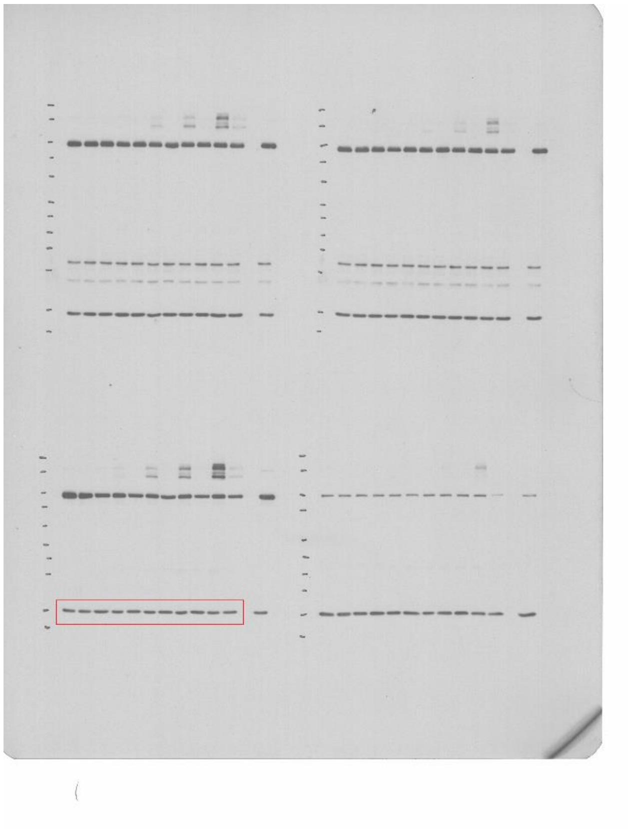

**Figure 1. Characterization of IWR1-POMA** (D) IWR1-POMA promoted a more complete degradation of β-catenin than IWR1 in HEK293 cells cultured with Wnt3A conditioned media.
