# Supplementary material for: Targeting tankyrase scaffolding in the β-catenin destruction complex by PROTAC overcomes the limitation of catalytic inhibitors in colorectal cancer
Source: eLife. 2026 Nov 4;14:RP109052. doi: 10.7554/eLife.109052 (PMC13734679; doi:10.7554/eLife.109052)
Supplement: Supplementary file 7 [file elife-109052-fig1-figsupp3-data1.zip › Figure 1-figure supplement 3-source data 2. Original files for western blot analysis displayed in Figure 1-S3B.pdf]

Fig1s3b_1_TNKS

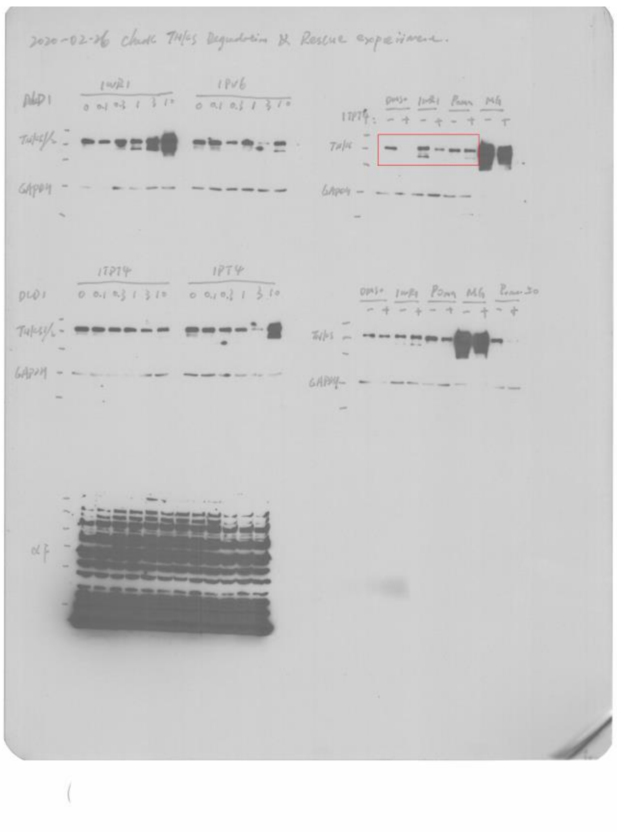

Fig1s3b_2_GAPDH

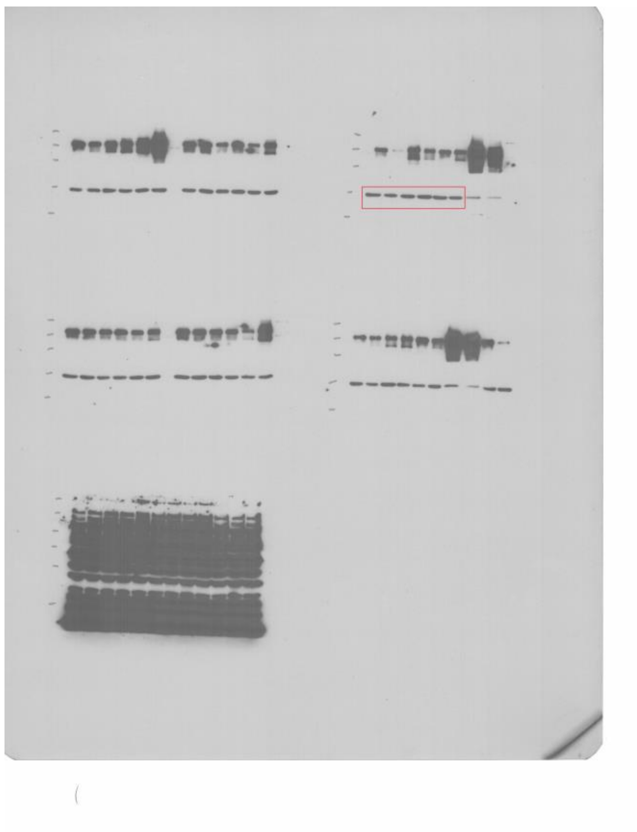

Figure 1S3. Additional characterization of IWR1-POMA (B) IWR1 (3 µM) and pomalidomide (3 µM) prevented the degradation of TNKS by IWR1-POMA (3 µM) in DLD-1 cells.
