# Supplementary material for: Targeting tankyrase scaffolding in the β-catenin destruction complex by PROTAC overcomes the limitation of catalytic inhibitors in colorectal cancer
Source: eLife. 2026 Nov 4;14:RP109052. doi: 10.7554/eLife.109052 (PMC13734679; doi:10.7554/eLife.109052)
Supplement: Supplementary file 7 [file elife-109052-fig1-figsupp3-data1.zip › Figure 1-figure supplement 3-source data 3. Original files for western blot analysis displayed in Figure 1-S3D.pdf]

Fig1s3d_1_HeLa_TNKS_GAPDH

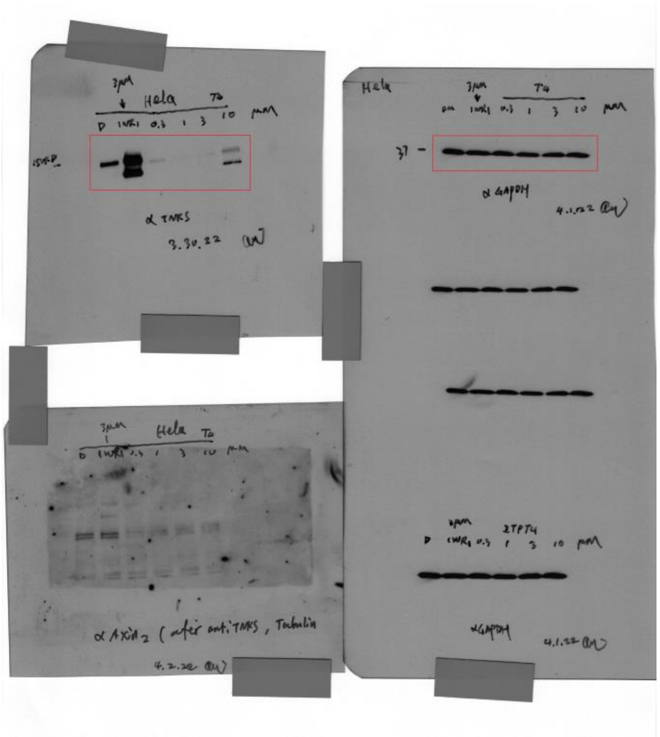

Fig1s3d_2_SW480_TNKS

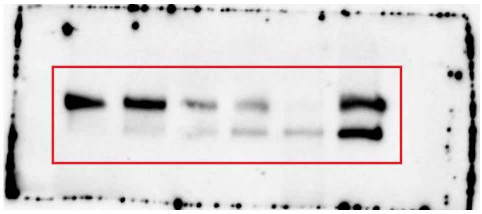

Fig1s3d_3_SW480_GAPDH

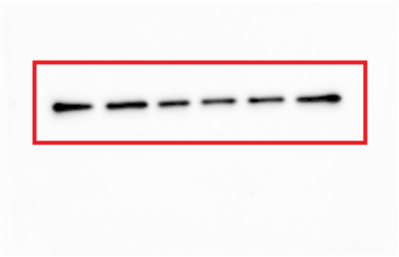

Fig1s3d_4_HT29_TNKS

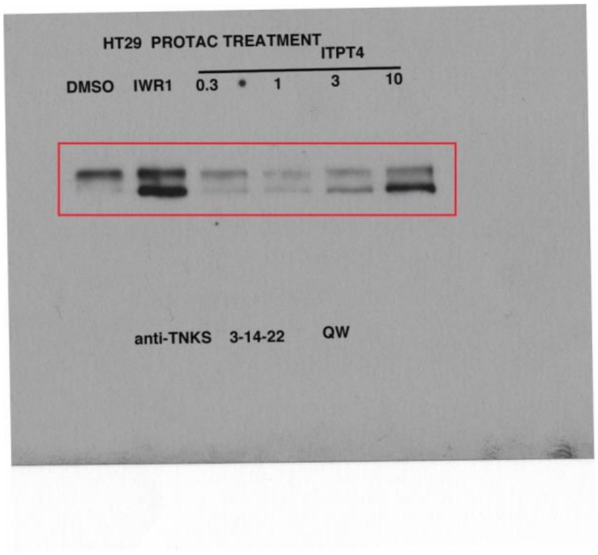

Fig1s3d_5_HT29_TUBA

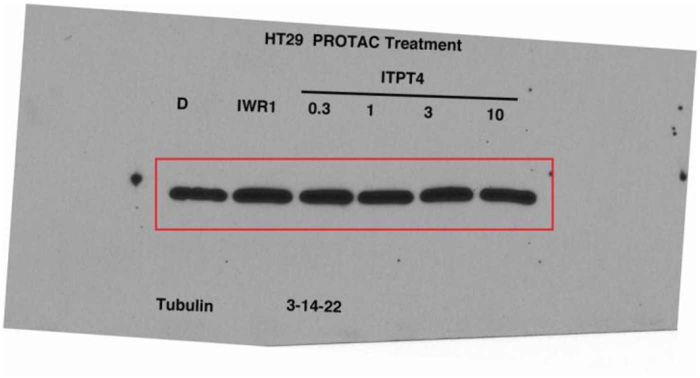

Figure 1S3. Additional characterization of IWR1-POMA (D) IWR1-POMA promoted TNKS degradation while IWR1 induced TNKS accumulation in SW480, HT-29 and HeLa cells.
