# Supplementary material for: Targeting tankyrase scaffolding in the β-catenin destruction complex by PROTAC overcomes the limitation of catalytic inhibitors in colorectal cancer
Source: eLife. 2026 Nov 4;14:RP109052. doi: 10.7554/eLife.109052 (PMC13734679; doi:10.7554/eLife.109052)
Supplement: Supplementary file 10 [file elife-109052-fig1-figsupp4-data1.zip › Figure 1-figure supplement 4-source data 1. Original files for western blot analysis displayed in Figure 1-S4A.pdf]

Fig1s4a_TNKS_GAPDH

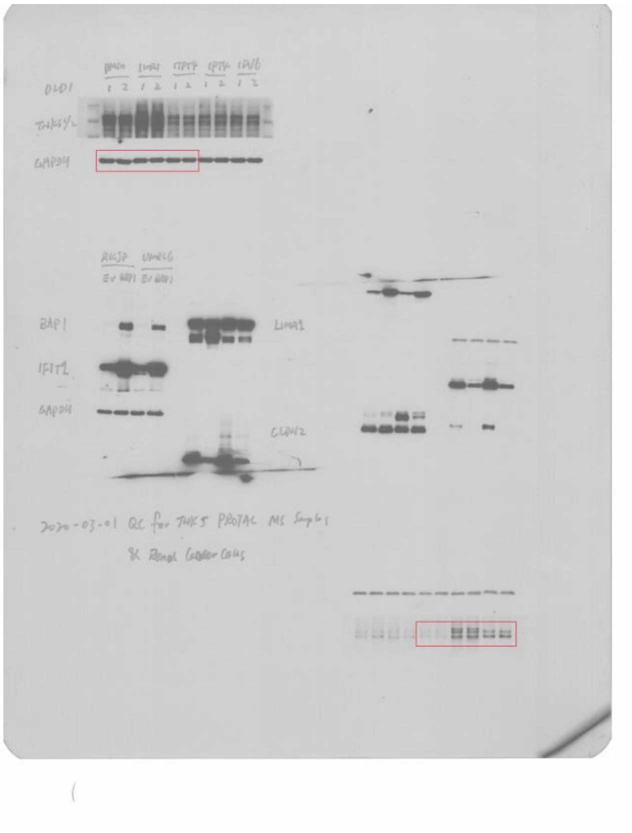

Figure 1S4. Proteomic analysis of DLD-1 cells treated with DMSO, IWR1 or IWR1-POMA (A) Western blot analysis of samples corresponding to Figure 1 E and 1F confirmed the accumulation of TNKS1/2 by IWR1 and the depletion by IWR1-POMA.
