# Supplementary material for: Targeting tankyrase scaffolding in the β-catenin destruction complex by PROTAC overcomes the limitation of catalytic inhibitors in colorectal cancer
Source: eLife. 2026 Nov 4;14:RP109052. doi: 10.7554/eLife.109052 (PMC13734679; doi:10.7554/eLife.109052)
Supplement: Supplementary file 12 [file elife-109052-fig2-figsupp1-data1.zip › Figure 2-figure supplement 1-source data 1. Original files for western blot analysis displayed in Figure 2-S1A.pdf]

Fig2s1a_1_TNKS

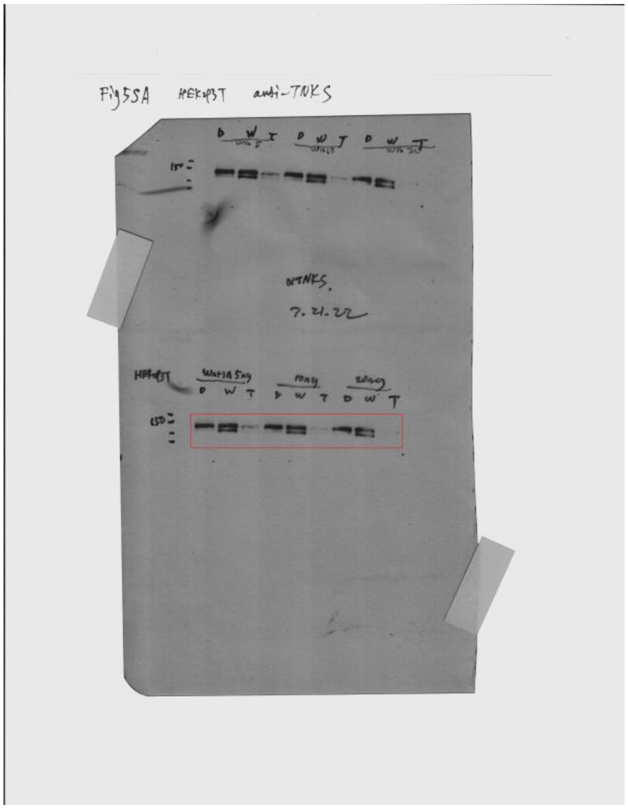

Fig2s1a_2_AXIN2

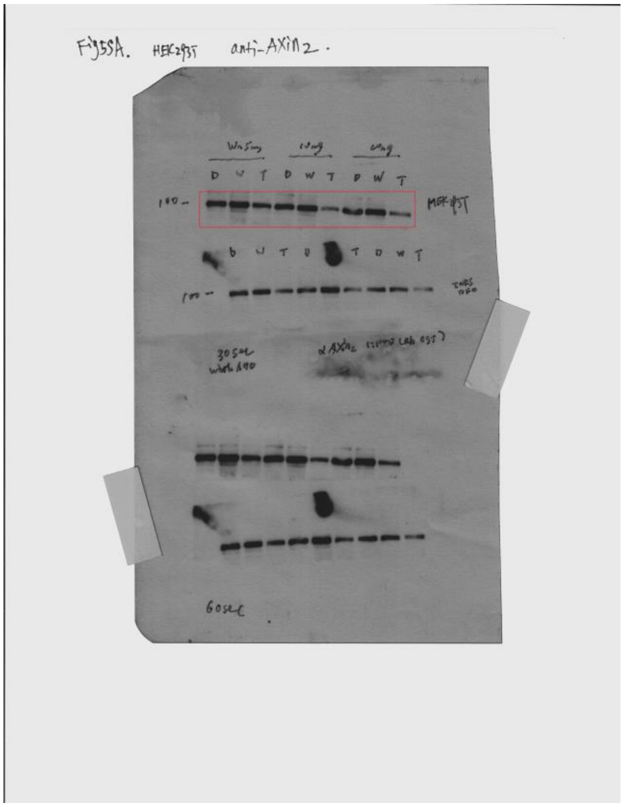

Fig2s1a_3_CTNNB1

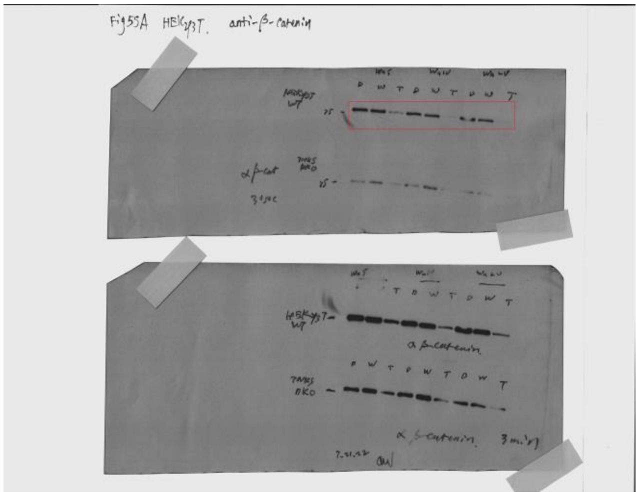

Fig2s1a_4_GAPDH

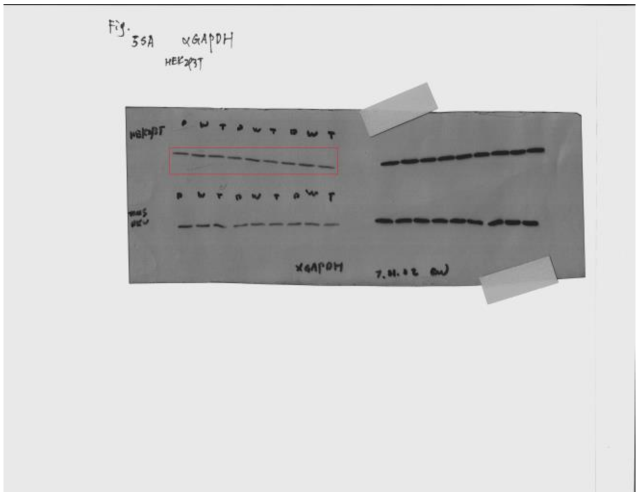

Figure 2S1. Degradation of TNKS by IWR1-POMA in 293T cells (A) 293T cells were transfected with different doses of Wnt3A plasmid and then treated with DMSO, IWR1 (3 μM) or IWR1-POMA (3 μM). The cytosolic fraction of the cell lysates was then examined by Western blot. IWR1-POMA promoted a more complete degradation of β-catenin than IWR1 and reduced the level of the WNT target AXIN2.
