# Supplementary material for: Targeting tankyrase scaffolding in the β-catenin destruction complex by PROTAC overcomes the limitation of catalytic inhibitors in colorectal cancer
Source: eLife. 2026 Nov 4;14:RP109052. doi: 10.7554/eLife.109052 (PMC13734679; doi:10.7554/eLife.109052)
Supplement: Supplementary file 12 [file elife-109052-fig2-figsupp1-data1.zip › Figure 2-figure supplement 1-source data 2. Original files for western blot analysis displayed in Figure 2-S1B.pdf]

Fig2s1b_1_TNKS_FLAG

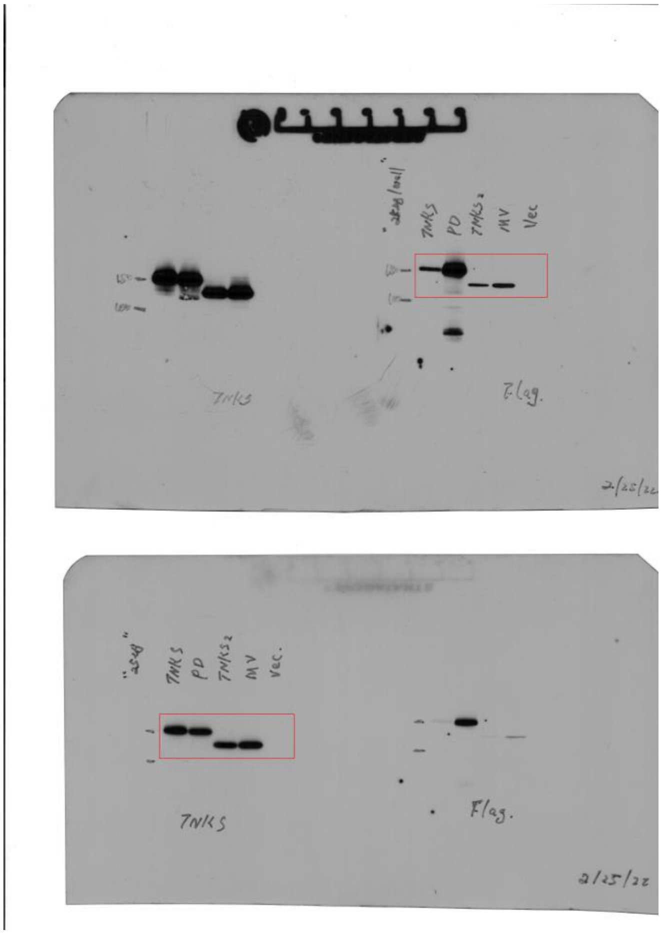

Fig2s1b_2_VCL

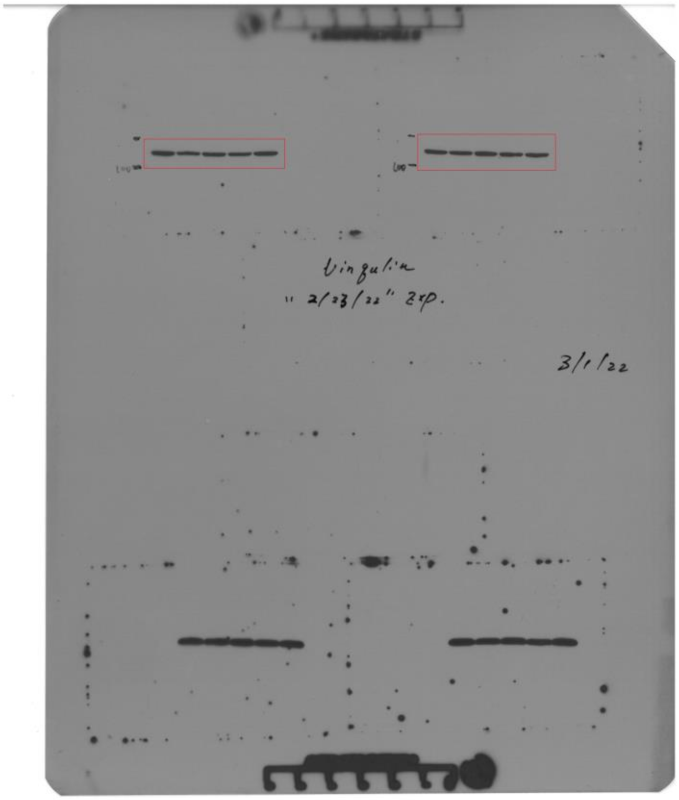

Figure 2S1. Degradation of TNKS by IWR1-POMA in 293T cells (B) Western blot analysis confirmed the lack of TNKS expression in 293T-TNKS1/2-DKO cells and validated the expression of FLAG-TNKS1, 3×FLAG-TNKS1-PD, FLAG-TNKS2 and FLAG-TNKS2-M1054V after transfection.
