# Supplementary material for: Targeting tankyrase scaffolding in the β-catenin destruction complex by PROTAC overcomes the limitation of catalytic inhibitors in colorectal cancer
Source: eLife. 2026 Nov 4;14:RP109052. doi: 10.7554/eLife.109052 (PMC13734679; doi:10.7554/eLife.109052)
Supplement: Supplementary file 14 [file elife-109052-fig5-data1.zip › Figure 5-source data 1. Original files for western blot analysis displayed in Figure 5D.pdf]

Fig5d_1_TNKS

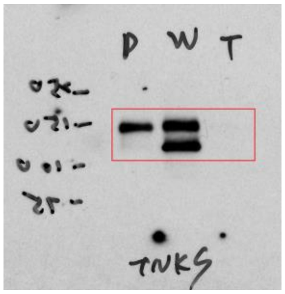

Fig5d_2_AXIN1

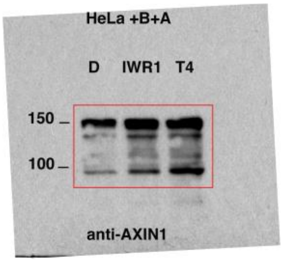

Fig5d_3_CTNNB1

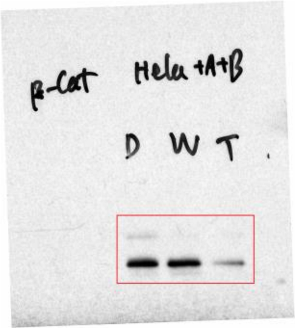

Fig5d_4_CTNNB1

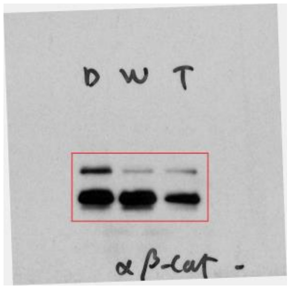

Fig5d_5_LEF

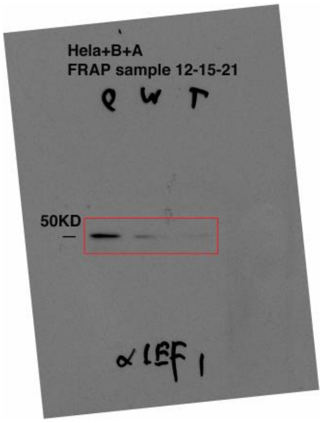

Fig5d_6_TUBA

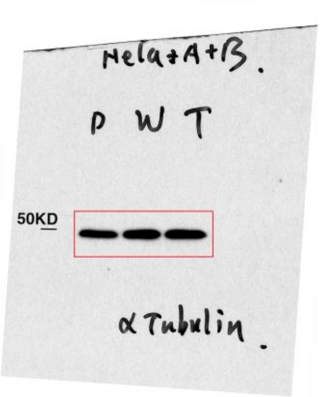

Figure 5. TNKS accumulation impeded the degradation of β-catenin (C) Western blot analysis of samples corresponding to Figure 5A confirmed the accumulation of TNKS by IWR1 and the depletion TNKS by IWR1-POMA. The deeper suppression of the Wnt/β-catenin signaling by IWR1-POMA is also supported by the reduced levels of total β-catenin.
