# Supplementary material for: Targeting tankyrase scaffolding in the β-catenin destruction complex by PROTAC overcomes the limitation of catalytic inhibitors in colorectal cancer
Source: eLife. 2026 Nov 4;14:RP109052. doi: 10.7554/eLife.109052 (PMC13734679; doi:10.7554/eLife.109052)
Supplement: Supplementary file 16 [file elife-109052-fig6-figsupp1-data1.zip › Figure 6-figure supplement 1-source data 1. Original files for western blot analysis displayed in Figure 6-S1D_1.pdf]

Fig6s1d_1_TNKS

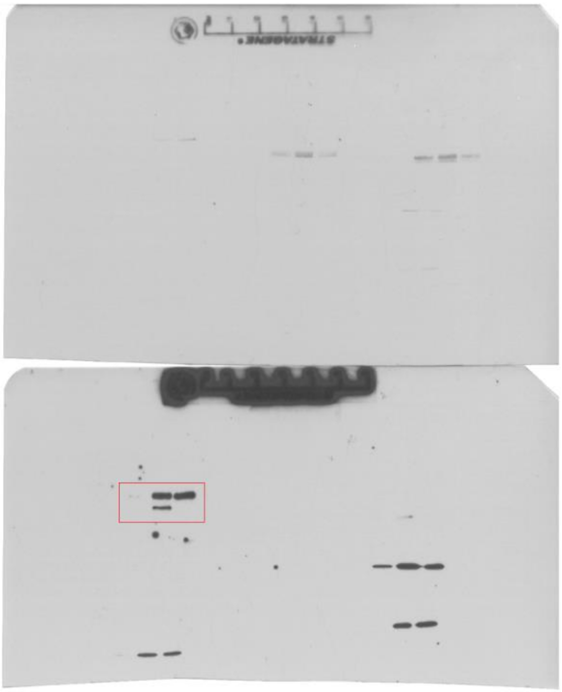

Fig6s1d_2_AXIN1

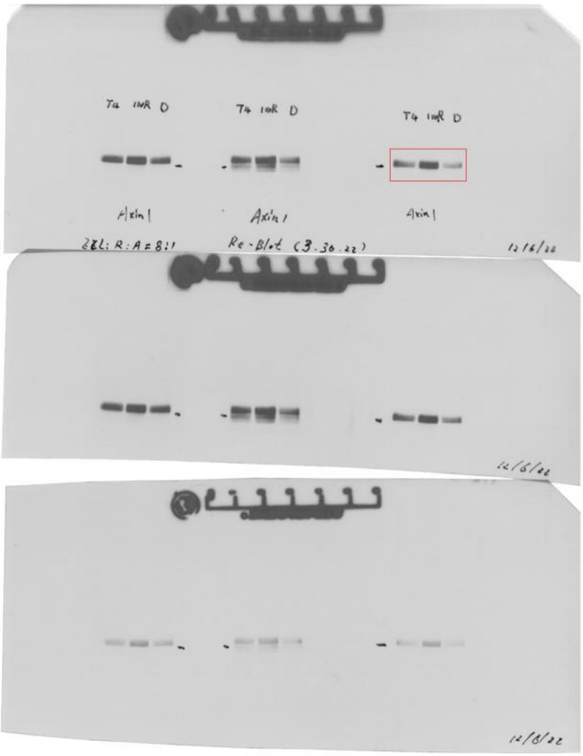

Fig6s1d_3_AXIN2

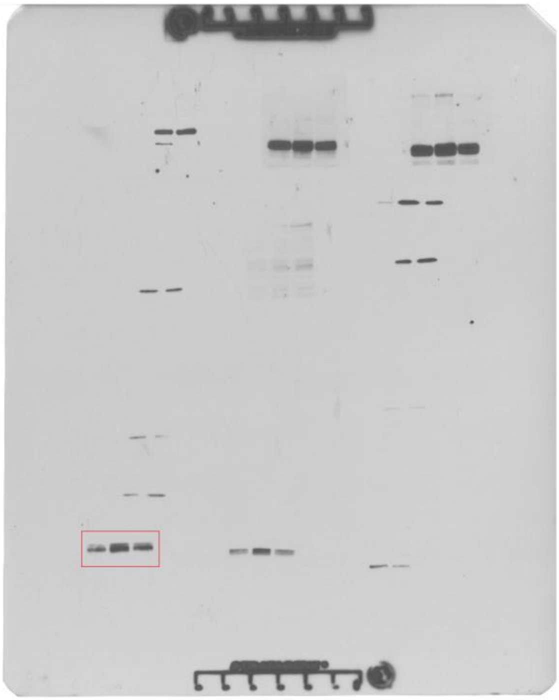

Fig6s1d_4_MYC

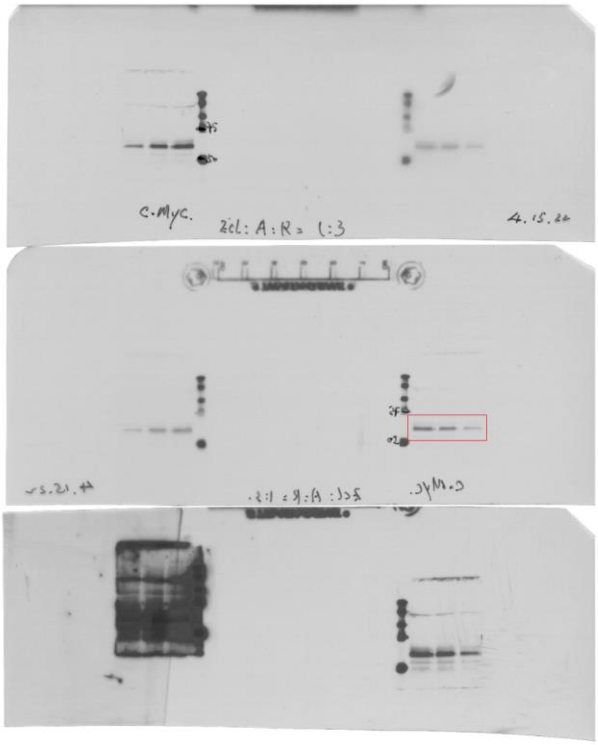

Figure 6S1. IWR1-POMA suppressed CRC growth through WNT inhibition (D) Western blot analysis confirmed that c-MYC, Aurora A, CDK4 and cyclin D1 responded to IWR1-POMA (3 μM) but not IWR1 (3 μM) in DLD-1 cells.
