# Supplementary material for: Targeting tankyrase scaffolding in the β-catenin destruction complex by PROTAC overcomes the limitation of catalytic inhibitors in colorectal cancer
Source: eLife. 2026 Nov 4;14:RP109052. doi: 10.7554/eLife.109052 (PMC13734679; doi:10.7554/eLife.109052)
Supplement: Supplementary file 18 [file elife-109052-fig6-figsupp2-data1.zip › Figure 6-figure supplement 2-source data 1. Original files for western blot analysis displayed in Figure 6-S2B.pdf]

Fig6s2b_1_TNKS

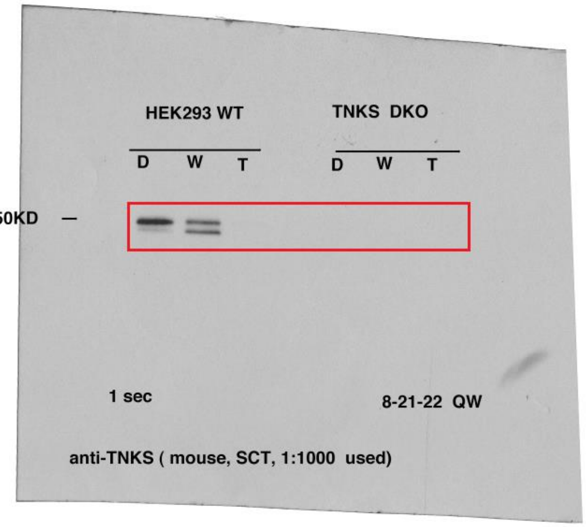


Fig6s2b_2_CTNNB1_GAPDH

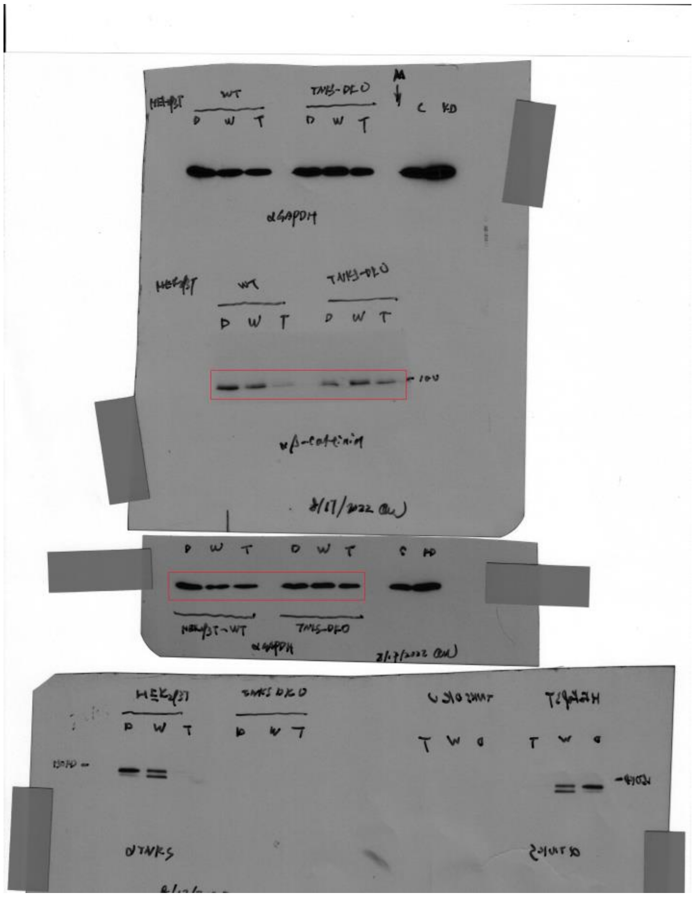


**Figure 6S2. IWR1-POMA suppressed CRC proliferation through on-target WNT inhibition** (B) IWR1-POMA (3 μM) reduced the cytosolic β-catenin level more effectively than IWR1 (3 μM) in 293T cells. The level of cytosolic β-catenin in 293T-TNKS1/2-DKO cells that lack both TNKS1 and TNKS2 did not change significantly with either drug treatment.
