# Supplementary material for: Targeting tankyrase scaffolding in the β-catenin destruction complex by PROTAC overcomes the limitation of catalytic inhibitors in colorectal cancer
Source: eLife. 2026 Nov 4;14:RP109052. doi: 10.7554/eLife.109052 (PMC13734679; doi:10.7554/eLife.109052)
Supplement: Supplementary file 18 [file elife-109052-fig6-figsupp2-data1.zip › Figure 6-figure supplement 2-source data 2. Original files for western blot analysis displayed in Figure 6-S2C.pdf]

Fig6s2c_1_GSPT1

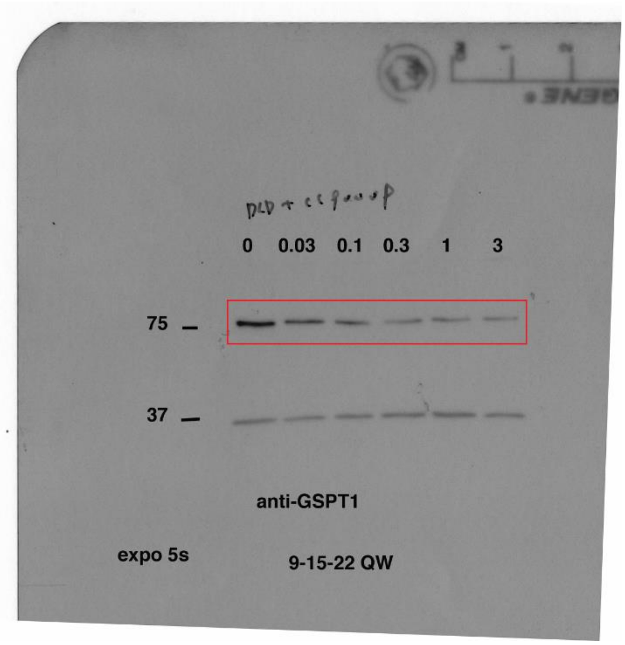

Fig6s2c_2_TNKS

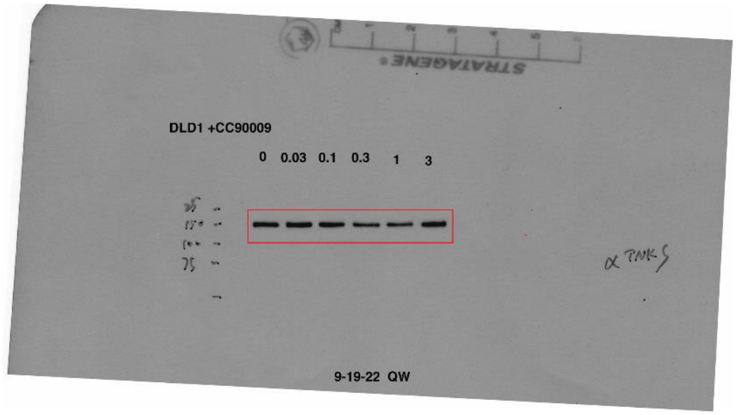

Fig6s2c_3_CTNNB1

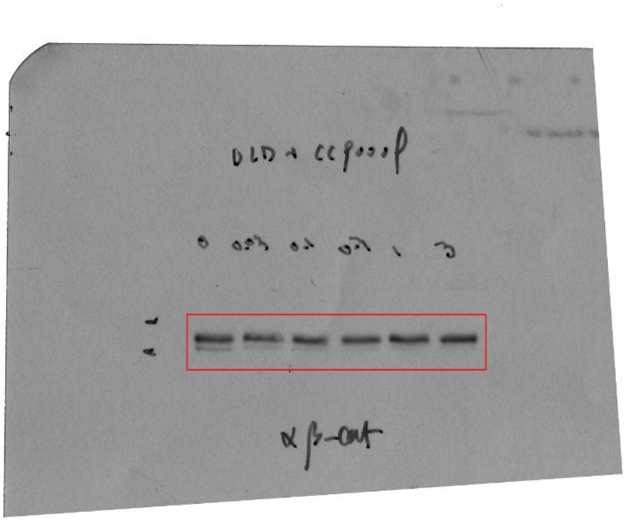

Fig6s2c_4_GAPDH

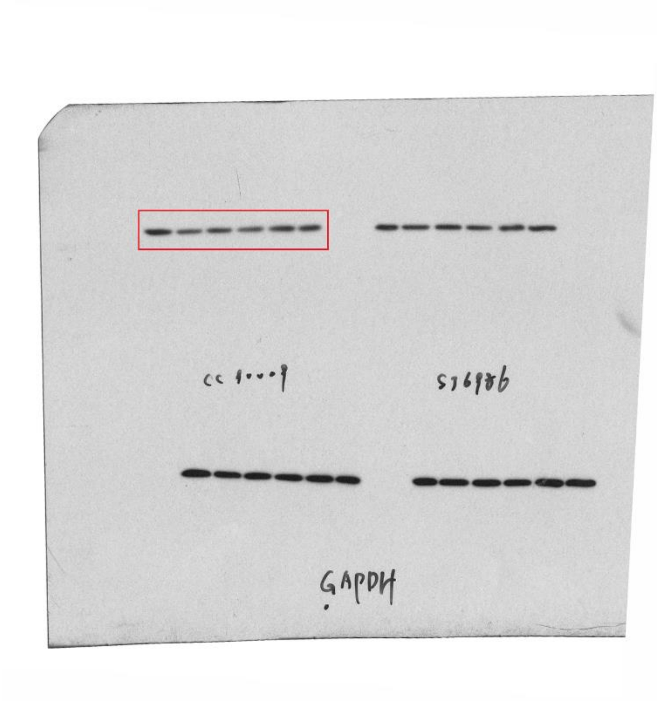

Figure 6S2. IWR1-POMA suppressed CRC proliferation through on-target WNT inhibition (C) CC-90009 induced GSPT1 degradation in DLD-1 cells but had little effect on TNKS. GSPT2 was not detectable by Western blot, which is consistent with the reported GSPT levels determined by quantitative proteomic analysis in this cell line—102,567 ppb for GSPT1 and 2,495 ppb for GSPT2 (https://www.ebi.ac.uk/gxa/experiments/E-PROT-18/Results).
