# Supplementary material for: Targeting tankyrase scaffolding in the β-catenin destruction complex by PROTAC overcomes the limitation of catalytic inhibitors in colorectal cancer
Source: eLife. 2026 Nov 4;14:RP109052. doi: 10.7554/eLife.109052 (PMC13734679; doi:10.7554/eLife.109052)
Supplement: Supplementary file 18 [file elife-109052-fig6-figsupp2-data1.zip › Figure 6-figure supplement 2-source data 3. Original files for western blot analysis displayed in Figure 6-S2D.pdf]

Fig6s2d_1_GSPT1

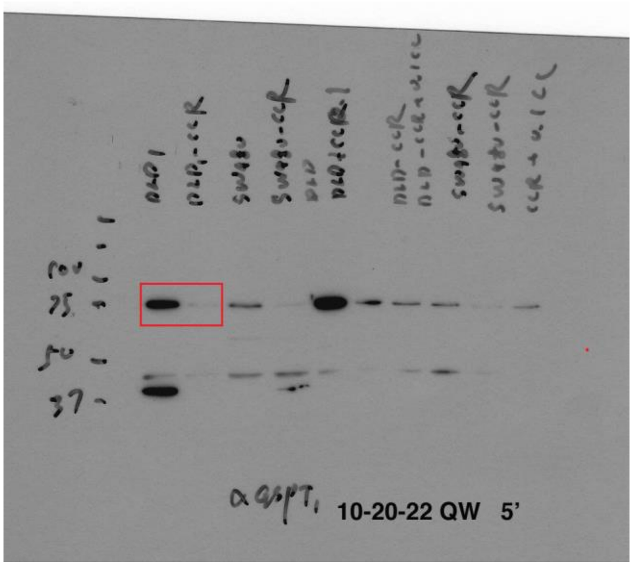

Fig6s2d_2_GAPDH

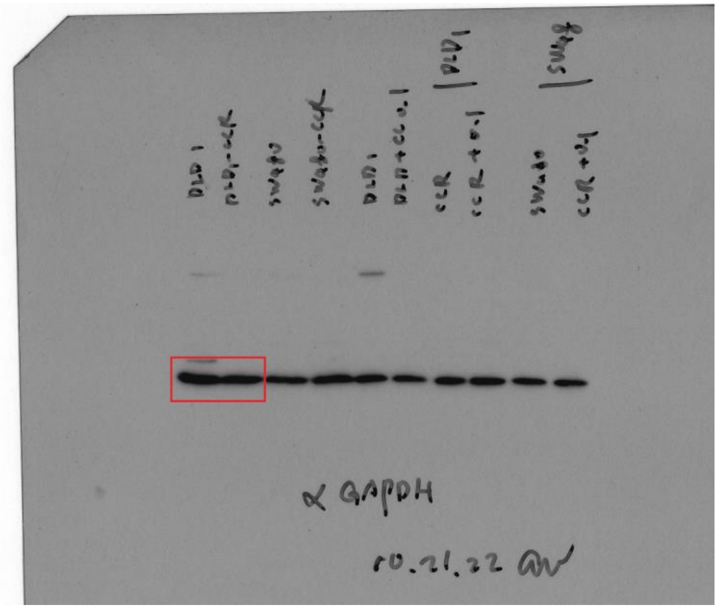

Figure 6S2. IWR1-POMA suppressed CRC proliferation through on-target WNT inhibition (D) DLD-1R cells obtained from cultivating DLD-1 cells with CC-90009 have a dramatically reduced level of GSPT1 expression meanwhile maintaining normal TNKS expression.
