# Supplementary material for: Targeting tankyrase scaffolding in the β-catenin destruction complex by PROTAC overcomes the limitation of catalytic inhibitors in colorectal cancer
Source: eLife. 2026 Nov 4;14:RP109052. doi: 10.7554/eLife.109052 (PMC13734679; doi:10.7554/eLife.109052)
Supplement: Peer review history

# Editor report: eLife Assessment

**DOI:** 10.7554/eLife.109052.4.sa0

**Authors:** Roel Nusse

**Affiliations:** Stanford University United States

**Keywords:** Convincing, Important

This **important** study reports the development of the first tankyrase degrader and demonstrates its enhanced ability to inhibit β-catenin signaling compared to conventional tankyrase inhibitors. The evidence supporting the conclusions is comprehensive and **convincing**, based on rigorous biochemical and cellular analyses. The findings will be of broad interest to researchers studying Wnt signaling, protein degradation, and cancer biology.

# Referee report: Reviewer #1 (Public review)

**DOI:** 10.7554/eLife.109052.4.sa1

[Editors' note: the second round of revision has been assessed by the Reviewing Editor without further input from the original reviewers. The authors have addressed the minor comments raised in the previous round of review.]

Summary:

This manuscript reports the discovery and characterization of the first bifunctional degrader of tankyrase. Notably, the tankyrase degrader exhibits stronger β-catenin inhibition and tumor growth suppression compared to conventional tankyrase inhibitors. Mechanistically, while tankyrase inhibitors stabilize tankyrase and promote Axin puncta formation-thereby impairing β-catenin degradation-the degrader avoids this effect, resulting in deeper suppression of β-catenin signaling. These findings suggest that targeted degradation of tankyrase offers a novel therapeutic strategy for β-catenin-driven cancers. Overall, this is a compelling study with significant translational potential.

Strengths:

(1) The manuscript presents a rigorous and well-executed study on a timely and impactful topic.

(2) The biochemical and cellular characterization of the tankyrase degrader is thorough, and the comparative analysis with tankyrase inhibitors is insightful.

(3) The finding that tankyrase stabilization by inhibitors may interfere with Axin function is novel and significant. It aligns with earlier observations (e.g., Huang 2009) that transient tankyrase overexpression can stabilize β-catenin independently of PAR domain activity.

(4) The use of TNKS1/2 knockout cells expressing catalytically inactive tankyrase to demonstrate β-catenin inhibitory activity of the tankyrase degrader is elegant.

(5) The finding that the tankyrase degrader has superior anti-proliferative effects in colorectal cancer models has important therapeutic implications.

Comments on previous version:

I had a favorable opinion of the manuscript in the first round of review. I don't have additional comments on the revised manuscript. The manuscript looks fine to me.

# Referee report: Reviewer #2 (Public review)

**DOI:** 10.7554/eLife.109052.4.sa2

Summary:

The ADP-ribosyltransferase tankyrase controls many biological processes, many of which are relevant to human disease. This includes Wnt/beta-catenin signalling, which is dysregulated in many cancers, most notably colorectal cancer. Tankyrase is a positive regulator of Wnt/beta-catenin signalling in that it counters the activity of the beta-catenin destruction complex (DC). Catalytic inhibition of tankyrase not only blocks PAR-dependent ubiquitylation and degradation of AXIN1/2, the central scaffolding protein in the DC, but also tankyrase itself. As a result, blocking tankyrase gives rise to tankyrase accumulation, which may accentuate its non-catalytic functions, which have been proposed to drive Wnt/beta-catenin signalling. Most tankyrase catalytic inhibitors have shown limited efficacy and substantial toxicity in vivo. By developing tankyrase-directed PROTACs, the authors aim to block both catalytic and non-catalytic functions of tankyrase, aspiring to achieve a more complete inhibition of Wnt/beta-catenin signalling. The successfully developed PROTAC, based on the existing catalytic inhibitor IWR1, IWR1-POMA, induces the degradation of both TNKS and TNKS2, blocks beta-catenin-dependent transcription without stabilising the DC in puncta/degradasomes, and inhibits cancer cell growth in vitro. Mechanistically, this points to a scaffolding role of tankyrase in the DC, at least under conditions of tankyrase catalytic inhibition, in line with previous proposals.

Strengths:

The study clearly illustrates the incentive for developing a tankyrase degrader, namely, to abolish both catalytic and non-catalytic functions of tankyrase. By and large, the study achieves these ambitions, and the findings support the main conclusions, although the statement that a more complete inhibition of the pathway is achieved requires corroboration. The proteomics studies are powerful. IWR1-POMA constitutes a very useful tool to re-evaluate targeting of tankyrase in oncogenic Wnt/beta-catenin signalling. The paired compounds will benefit investigations of tankyrase scaffolding functions across many different biological systems controlled by tankyrase. The findings are exciting.

Comments on previous version:

I thank the authors for responding to the queries raised in the original review, most of which have now been addressed. This further strengthens this well-conducted study and well-presented manuscript. I congratulate the authors for this interesting and insightful work.

# Referee report: Reviewer #3 (Public review)

**DOI:** 10.7554/eLife.109052.4.sa3

In this manuscript, Wang et al employ a chemical biology approach to investigate the differences between the enzymatic and scaffolding roles of tankyrase during Wnt β-catenin signalling. It was previously established that, in addition to its enzymatic activity, tankyrase 1/2 also plays a scaffolding function within the destruction complex, a property conferred by SAM-domain-dependent polymerization (PMID: 27494558). It is also known that TNKS1/2 is an autoregulated protein and that its enzymatic inhibition leads to accumulation of total TNKS proteins and stabilization of Axin punctae (through the scaffolding function of TNKS1/2), leading to rigidification of the DC and decreased β-catenin turnover. The authors surmised that this could, in part, explain the limited efficacy of TNKS1/2 catalytic inhibition for the treatment of colorectal cancers. To test this hypothesis, they evaluated a series of PROTAC molecules promoting the degradation of TNKS1/2 to block both the catalytic and scaffolding activities. They show that IWR1-POMA (their most active molecule) promotes more efficient suppression of beta-catenin-mediated transcription and is more active in inhibiting colorectal cancer cell and CRC patient-derived organoids growth. Mechanistically, the authors used FRAP to demonstrate that catalytic inhibitors of TNKS led to a reduced dynamic assembly of the DC (rigidification), whereas IWR1-POMA did not affect the dynamics.

Overall, this is an interesting study describing the design and development of a PROTAC for TNKS1/2 that could have increased efficacy where catalytic inhibitors have displayed limited activity. Knowing the importance of the scaffolding role of TNKS1/2 within the destruction complex, targeting both the catalytic and scaffolding roles certainly makes sense. The manuscript contains convincing evidence of the different mechanisms of the PROTAC vs catalytic inhibitors. Some additional efforts to quantify several of the experiments and to indicate the reproducibility and statistical analysis would strengthen the manuscript. Ultimately, it would have been great to evaluate the in vivo efficacy of IWR1-POMA in an in vivo CRC assay (APCmin mice or using PDX models); however, I realize that this is likely beyond the scope of this manuscript.

# Referee report: Reviewer #4 (Public review)

**DOI:** 10.7554/eLife.109052.4.sa4

From the Reviewing Editor:

This important study reports the development of the first PROTACs targeting the ADP-ribosyltransferases tankyrase 1 and 2, with the goal of inhibiting Wnt/β-catenin signaling more completely than is possible with catalytic tankyrase inhibitors. The work addresses a significant limitation of existing tankyrase inhibitors: although catalytic inhibition stabilizes AXIN1/2 and suppresses Wnt signaling, it also stabilizes tankyrase itself, potentially enhancing non-catalytic scaffolding functions and promoting accumulation of degradasome-like puncta.

The evidence is convincing. The authors use appropriate and well-validated approaches, including chemical biology, cellular assays, and proteomic profiling, to show that PROTAC-mediated degradation of tankyrase avoids tankyrase accumulation while still stabilizing AXIN and inhibiting Wnt/β-catenin signaling. The data support the conclusion that degradation of tankyrase can separate pathway inhibition from the confounding effects of stabilized tankyrase protein and may therefore offer advantages over conventional catalytic inhibitors.

A strength of the study is the clear mechanistic comparison between tankyrase degradation and catalytic inhibition. The manuscript provides convincing evidence that the PROTAC and catalytic inhibitors act through distinct mechanisms, with the PROTAC targeting both catalytic and scaffolding roles of tankyrase. The study is well conducted and clearly presented, and the authors have addressed most concerns raised during review.

A remaining limitation is that the therapeutic potential of the compound is not tested in vivo, for example in APC-mutant colorectal cancer models, APCmin mice, or patient-derived xenografts. Such experiments would strengthen claims about practical efficacy, although they are not essential for the main mechanistic conclusions of the manuscript.

Overall, this is an important and insightful contribution. It advances the tankyrase and Wnt signaling fields by providing a new chemical strategy to suppress tankyrase function more completely than catalytic inhibition alone, and it offers a useful framework for future therapeutic exploration of tankyrase degradation.

# Author comment: Author response

**DOI:** 10.7554/eLife.109052.4.sa5

**Authors:** Qian Wang, Liping Li, Lin You, Shuai Wang, Lei Han, Bingnan Wang, Liping Yao, Yesu Addepalli, Yong Lu, Ilgen Mender, Ann M Flusche, Chiho Kim, Nageswari Yarravarapu, Andrew Lemoff, Lawrence Lum, Jerry W Shay, Yonghao Yu, Chuo Chen

**Affiliations:** University of Texas Southwestern Medical Center Dallas United States; The University of Texas Southwestern Medical Center Dallas United States; The University of Texas Southwestern Medical Center Dallas United States; The University of Texas Southwestern Medical Center Dallas United States; The University of Texas Southwestern Medical Center Dallas United States; The University of Texas Southwestern Medical Center Dallas United States; The University of Texas Southwestern Medical Center Dallas United States; The University of Texas Southwestern Medical Center Dallas United States; The University of Texas Southwestern Medical Center Dallas United States; The University of Texas Southwestern Medical Center Dallas United States; The University of Texas Southwestern Medical Center Dallas United States; The University of Texas Southwestern Medical Center Dallas United States; The University of Texas Southwestern Medical Center Dallas United States; The University of Texas Southwestern Medical Center Dallas United States; University of Texas Southwestern Medical Center Dallas United States; The University of Texas Southwestern Medical Center Dallas United States; The University of Texas Southwestern Medical Center Dallas United States; The University of Texas Southwestern Medical Center Dallas United States

The following is the authors’ response to the previous reviews

We thank the Reviewers for the favourable feedback. There is no additional comments from Reviewers 1, 3, and 4, and we address the minor concerns from Reviewer 2 as follows.

(1) I appreciate the authors acknowledge that testing the physical properties of the degradasome puncta is necessary to explore whether they indeed represent condensates. The term "condensates" implies liquid-liquid phase separation (rightly or wrongly). However, this question has not yet been resolved in the case of degradasomes. I therefore suggest the term "condensates" to be avoided. A simple morphological description as "puncta" may suffice.

We have revised our manuscript to state: “The DC has been proposed to exist as biomolecular condensates.” Additionally, we have included a time-lapse image showing that AXIN1-GFP puncta exhibit dynamic fusion behaviour in cells (Fig. S8), suggesting that the DC may be liquid-like, at least with AXIN1 overexpression.

(2) I thank the authors for including the additional data comparing tankyrase binding by IWR and IWRPOMA. I agree that using the BRET signal of IWR-POMA is informative. Adding the IC_50_ values directly to the figure panels (S3E, S3G) would help the reader to quickly assess binding. The comparison between these two panels is insightful.

Added.

(3) Regarding the use of the terms TNKS, TNKS1 and TNKS2, if the authors would like to use the name "TNKS" to refer to both paralogues collectively, can this please be specified early in the manuscript to limit confusion with the official gene name "TNKS", which of course only refers to one paralogue? Regarding the use of the terms TNKS, TNKS1 and TNKS2, if the authors would like to use the name "TNKS" to refer to both paralogues collectively, can this please be specified early in the manuscript to limit confusion with the official gene name "TNKS", which of course only refers to one paralogue?

We now specify in the Introduction that TNKS1/2 are encoded by *TNKS/TNKS2*, and are collectively referred to as TNKS in this manuscript.
